# Autofluorescence of MDA-modified proteins as an *in vitro* and *in vivo* probe in oxidative stress analysis

**DOI:** 10.1007/s13238-014-0052-1

**Published:** 2014-04-03

**Authors:** Min Qiang, Yajie Xu, Yang Lu, Yingge He, Chanshuai Han, Ying Liu, Rongqiao He

**Affiliations:** 1 State Key Laboratory of Brain and Cognitive Science, Institute of Biophysics, Chinese Academy of Sciences, Beijing, 100101 China; 2 Key Laboratory of Mental Health, Institute of Psychology, Chinese Academy of Sciences, Beijing, 100101 China; 3 University of Chinese Academy of Sciences, Beijing, 100049 China

**Dear Editor,**

Malondialdehyde (MDA) is regarded as a biomarker for oxidative stress (Del Rio et al., 2005; Stancliffe et al., 2011), and is generated in the oxidative degradation process of polyunsaturated lipids (Stancliffe et al., 2011). This compound, a reactive electrophile species, contains two aldehyde groups and exhibits cytotoxic effects. As a product of lipid peroxidation, MDA was found to accumulate during many pathophysiological processes (Miller et al., 2011), most importantly in patients with cardiovascular disease, diabetes mellitus (Su and He, 2014) and neurodegeneration (Bagatini et al., 2011), where blood MDA levels are markedly increased (Sanyal et al., 2009). Therefore, to trace MDA and MDA-modified protein (*in vivo* and *in vitro*), and to investigate its role in oxidative stress, it is important to understand the etiology of cardiovascular disease and neurodegenerative disease.

Malondialdehyde is an active modifying agent of proteins both *in vitro* and *in vivo* (Foettinger et al., 2006; Weismann et al., 2011), causing protein aggregation and amyloid deposition (Allen et al., 1984). MDA-protein aggregates continuously enter cells within our body, especially under conditions of oxidative stress, which can be induced in various pathological conditions such as cardiovascular or neurodegenerative diseases. While the reaction with MDA results in the formation of covalent protein adducts that emit fluorescence (Kikugawa et al., 1984; Xu et al., 2012), the characteristics of these MDA-protein adducts and its utilization as a fluorescent probe have not been well studied so far. Here, we exploited this trait of MDA and modified bovine serum albumin (BSA), a protein that is susceptible to MDA-modification, to generate MDA-modified BSA (mBSA) that could be employed as a fluorescent probe in live cells.

In order to investigate the effect of MDA-modification on protein aggregation, we generated mBSA and analyzed its behaviour by SDS-PAGE. After incubation of BSA with different concentrations of MDA, and subsequent removal of un-incorporated MDA by ultrafiltration, mBSA was analyzed by 12% SDS-PAGE and Coomassie brilliant blue staining of the gel (Fig. 1). Polymers of mBSA could be observed over the period of 24 h in a MDA concentration-dependent manner (Fig. 1A). Further experiments of BSA incubated with 2 mmol/L MDA for different time intervals showed that mBSA formed polymers in a time-dependent fashion (Fig. 1B). The formation of polymers was confirmed by transmission electron microscopy. The modified proteins appeared to form a polymeric structure (Fig. 1C and 1D), while no polymers were visible in the unmodified BSA samples (Fig. 1E). MDA modification not only induced polymerization of BSA, but also endowed the modified protein with fluorescent properties (Fig. S1). The fluorescence intensity of mBSA exhibited a linear correlation with the concentration of MDA, ranging from 0.5 mmol/L to 4 mmol/L (Fig. S1A). The reaction of MDA with BSA was completed within ~48 h, as measured by the changes in both emission and excitation intensity (Fig. S1B and S1D) and quantum yield (Fig. S1C). Hence, BSA samples incubated with MDA for 48 h were used for further cell experiments. Compared with the emission intensity of mBSA (Fig. S2A), fluorescence detected for BSA alone, MDA and PBS was negligible (Fig. S2B, S2C and S2D).

**Figure 1:**
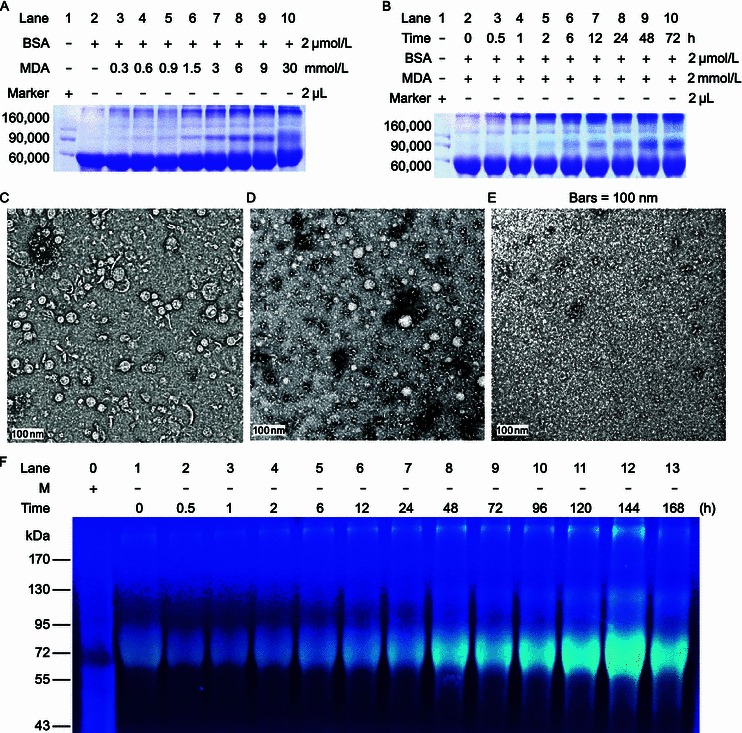
**Modification with malondialdehyde results in aggregation of BSA (mBSA)**. SDS-PAGE (12%) analysis followed by Coomassie brilliant blue staining of BSA incubated with different concentrations of MDA as indicated (A), and BSA incubated with 2 mmol/L MDA for different time intervals (B). Aliquots of reaction products at 24 h (C) and 48 h (D) were used to measure particle sizes by transmission electron microscopy. Unmodified BSA incubated for 48 h was used as control (E). The mBSA incubated for different time intervals was analyzed in gel, and was visualized under an ultraviolet light (F), the reaction conditions were identical as those used for panel B. M, marker

The maximum emission wavelength of mBSA was determined to be ~465 nm (Fig. S3A), and the maximum excitation ~400 nm, though two additional excitation wavelengths were detected (235 nm, 260 nm) (Fig. S3B). Three-dimensional fluorescence spectra, in which the fluorescence intensity is presented as a function of the excitation wavelength on one axis and the emission wavelength on the other, confirmed these measurements (Fig. S3C). Unmodified BSA shown none of fluorescence in three-dimensional fluorescence spectra (Fig. S3D). The fluorescence quantum yield and the fluorescent life of mBSA were determined to be 0.16 s and 3.51 × 10^−9^ s, respectively.

In summary, the MDA derivative of BSA exhibits a specific fluorescence emission at 465 nm, whilst MDA itself does not fluoresce at all; its fluorescent quantum yield is as high as 0.163, indicating that it has the potential of a probe with excellent signal to noise ratio.

Thus fluorescent protein bands on an SDS-PAGE gel could be visualized easily under UV light, without the need for Coomassie brilliant blue staining, as shown in Fig. S4A. The unmodified BSA used as control showed no protein bands under the UV light radiation (Fig. S4B), while bands were visible after Coomassie brilliant blue staining (Fig. S4C). Furthermore, a fluorescent signal was observed for both mBSA monomers as well as polymers. To test if other proteins can be labeled fluorescently using MDA with similar efficiency, alpha-synuclein, the primary component in Lewy Body found in brains of Parkinson’s disease patients (Shi and Wang, 2013) was modified. Similar to mBSA, alpha-synuclein was visualized on SDS-PAGE gel upon exposure to UV light (Fig. S5).

The above characteristics of MDA-derived proteins clearly make MDA an excellent candidate as a fluorescent biomarker applied to studies in living cells. To determine the optimal pH value for MDA derivatives, fluorescence intensity of mBSA was analyzed, and a maximal emission intensity detected at around pH 7.0 (Fig. S6A), making it suitable as a fluorescent probe *in vitro* and *in vivo*. To further investigate the effect of the modified protein on vital cellular functions (Yu et al., 2014), a cell viability assay was utilized (Liu et al., 2011). Whilst BSA showed a low inhibition of cell viability of BV-2 cells overall, with about 70% compared to the control (viability taken as 1), the viability did decrease with increasing concentrations of mBSA (0–15 μmol/L) (Fig. S6B). In the presence of unmodified BSA, cells exhibited a similar decrease in viability, suggesting that the toxicity of mBSA itself is not of major concern when used as a fluorescent probe for cellular studies.

Fluorescence signal was used to trace the internalization of mBSA. To trace mBSA in live cells, 1.5 × 10^3^ BV-2 cells were seeded onto a cover-glass bottom dish. After 12 h of incubation, cells were treated with 7.5 μmol/L mBSA for 2 h, and were observed immediately by confocal laser scanning microscopy. As shown in the merged photo presented in Fig. S7, the fluorescence signal of mBSA inside the BV-2 cells was observed as blue light, with no signal detected from cells incubated with unmodified BSA (control). More specifically, mBSA was found predominantly within the cytoplasm upon entering the BV-2 cells, which is in agreement with the fact that BSA is larger than the exclusion size of the nuclear pore complex. Subsequently, the precise *in-situ* location of mBSA was analyzed by staining with Dil, a lipophilic tracer for live cells (Fig. 2A). The cells cultured in the presence of mBSA were washed with fresh medium before being stained with Dil for 2 h. MDA-modified BSA was shown to be located intracellularly. In cells cultured with unmodified BSA, only red fluorescence of the Dil was observed (Fig. S8). Together, these results provide further proof that fluorescent MDA derivatives can be employed to trace proteins inside live cells.

**Figure 2:**
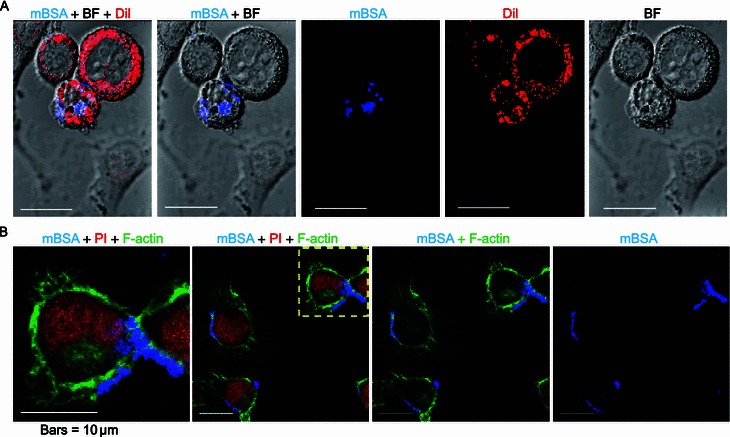
**Live and fixed BV-2 cells in the presence of MDA-modified BSA with lipophilic tracer Dil or F-actin dye Phalloidin**. BV-2 cells were cultured in the presence of mBSA (final concentration 7.5 μmol/L) in DMEM medium for 24 h, and then stained with lipophilic tracer Dil (red) for 2 h. Most of the signal of mBSA (blue) overlapped with that of Dil (A). BV-2 cells were fixed, and then stained with F-actin dye Phalloidin and propidium iodide (PI) for nuclei after 24 h incubation with mBSA (B). The fluorescence of mBSA (blue), F-actin (green) and nuclei (red) are shown using a confocal microscopy as indicated. ‘BF’ represents ‘bright field’. Bars = 10 μm

It is well-established that F-actin participates in the endocytosis process (Smythe and Ayscough, 2006). If F-actin also plays a role in the endocytosis of mBSA polymers, both should be found to co-localize in the cytoplasm. To this end, we analyzed the intracellular location of mBSA upon cell entry using immunofluorescence staining. As shown in Fig. 2B, vesicle-like fluorescent speckles (azure) that co-localized with both F-actin (green) and mBSA (blue) were observed in the BV-2 cells after 24 h of mBSA treatment. In contrast, cells treated with unmodified BSA only exhibited red nuclear staining, and green fluorescence for F-actin filaments (Fig. S9). This suggests that mBSA is able to enter BV-2 cells, and that an actin-related process might be involved in the uptake of this protein. In summary, the fluorescent signal of MDA adducts is a convenient tool for the study of both extracellular and intracellular translocation.

In conclusion, we developed a protocol for the modification of proteins with MDA, which not only induces protein polymerization, but also endowed the protein with a fluorescence property. This trait enables the visualization of the modified protein under UV light. Using lipophilic tracer Dil and markers for F-actin, we observed translocation of mBSA into microglia BV-2 cells and co-localization of mBSA with F-actin. Thus, MDA modifications can be used safely as fluorescent tools to trace the location of the modified proteins in cellular studies, without interfering with the proper functioning of vital cellular functions.

## Electronic supplementary material

Below is the link to the electronic supplementary material.

Supplementary material 1 (PDF 118 kb)

Supplementary material 2 (PDF 983 kb)
