# Supplementary material for: Autofluorescence of MDA-modified proteins as an in vitro and in vivo probe in oxidative stress analysis
Source: Protein Cell. 2014 Apr 3;5(6):484–7. doi: 10.1007/s13238-014-0052-1 (PMC4026423; doi:10.1007/s13238-014-0052-1)
Supplement: Supplementary file 1 — Supplementary material 1 (PDF 118 kb) [file 13238_2014_52_MOESM1_ESM.pdf]

Supplementary Data 1

Supplementary Figure Legends

Figure S1. Quality control of fluorescent product of malondialdehyde-modified BSA.

BSA (final concentration 45 μM) was incubated with MDA at different concentrations (0, 0.8, 1.5, 2.0, 2.5, 3.0, 4.0, 5.2, 6.0, 10.0 and 12 mM) in 10 mM PBS buffer (pH 7.4) at 37°C for 24 h. Spectra were measured at different time points for both emission (diluted 200-fold, λ_{ex}=405 nm, λ_{em}: 410-700 nm) and absorption (diluted 200-fold, λ_{em}=460 nm, λ_{ex}: 200-700 nm). The maximum fluorescent intensities of both emission spectra and absorption spectra are shown in panel (**a**). Under the same conditions used, 2 mM MDA was chosen to react with 45 μM BSA, and the fluorescence emission and absorption intensities were measured at different time points (panel **b**). Changes in fluorescent quantum yields of mBSA at different time points were detected using a Horiba Jobin Yvon Fluorolog-3 spectrofluorometer (panel **c**). The same data from panel **b** were plotted using the Tsou's method to analyze kinetic formation of fluorescence. The increase displayed a biphasic behaviour, showing a fast and slow phase (panel **d**). The first order rate constant of the fast phase was $2.13\times10^{-5} \cdot s^{-1}$, and the slow one was $9.84\times10^{-6} \cdot s^{-1}$.

Figure S2. Emission fluorescence of MDA-modified BSA and control.

Changes in the emission fluorescence of mBSA (identical conditions were chosen as described in **Fig. S1**) are shown (panel **a**). The emission fluorescence for controls are shown for BSA alone (panel **b**), MDA (panel **c**) and PBS (panel **d**). 45 μM BSA was incubated with 2 mM MDA for 0-168 h and diluted to 0.5 μM, before fluorescence measurement. Fluorescence for 'mBSA', 'MDA' and 'PBS' was similar to background

reading.

Figure S3. Two- and three-dimensional fluorescent spectra of MDA-modified BSA.

The emission and excitation spectra of MDA-modified BSA (final concentration 0.25 μM) were measured at 37°C using a Hitachi F-4500 spectrofluorometer after 48 h of incubation. Both emission spectrum (λ_{ex} = 405 nm, with a slit of 3 nm) (panel **a**) and excitation spectrum (λ_{em} = 460 nm, with a slit of 3 nm) (panel **b**) of mBSA incubated with 2 mM MDA for 48 h were measured at 37°C, pH 7.4. Three-dimensional fluorescence of mBSA (panel **c**) and that of BSA alone as control (panel **d**) were measured under identical conditions.

Figure S4. Visualization of fluorescence of MDA-modified BSA protein bands on SDS-PAGE.

Conditions for BSA modification were identical to those described in **Fig. 1**. Aliquots were run at 12% SDS-PAGE at different time points, followed by Coomassie brilliant blue staining for visual control of protein presence (panel **a**). Unmodified BSA was detected under UV light in a Gel image analyzer (ChampGel 5000, Sagecreation, China) (panel **b**) and then stained by Coomassie brilliant blue for verification (panel **c**).

Figure S5. Visualization of fluorescence of MDA-modified alpha-synuclein (mAS) bands on SDS-PAGE.

Conditions for alpha-synuclein modification were identical to those described in **Fig. 1**, mAS samples were run on a 15% SDS-PAGE. mAS was visualized in gel under an UV light using a Gel image analyzer (ChampGel 5000, Sagecreation, China) (panel **a**) and followed by Coomassie brilliant blue staining (panel **b**). Unmodified alpha

synuclein could not be visualized under UV light (panel **c**), and gel was stained by Coomassie brilint blue (panel **d**) as control.

Figure S6. pH-dependent changes in the fluorescence of MDA-modified, and impact on cell viability of mBSA in the culture media.

Conditions used were identical to those for **Fig. S3**, except that measurements were made in buffers of different acidities (from pH 3 to pH 13) (panel **a).** Viability of BV-2 cells in the presence of mBSA at the concentrations indicated, compared to unmodified BSA, was determined by colorimetric MTT assay (panel **b**). Viability of untreated control cells was arbitrarily set to 1, and the fold-increase in absorbance was calculated against control. mBSA refers to MDA-modified BSA.

Figure S7. Tracing mBSA in live BV-2 cells.

Live BV-2 cells were treated with mBSA (final concentration 7.5 μM) in DMEM medium for 24 h, and observed using a confocal microscope (fluorescence shown in panel **a,** bright field shown in panel **b**) (excitation wavelength: 405 nm). BV-2 cells treated with unmodified BSA for 24 h were used as negative control (panel **c**). mBSA refers to MDA-modified BSA; BF refers to bright field. Bars=10 μm.

Figure S8. Immunocytofluorescence imaging of BV-2 cells in the absence of MDA-BSA.

Conditions used were identical to those in **Fig. 2a**. Cells were treated with unmodified BSA for 24 h, and subsequently stained with lipophilic tracer Dil (red color). Bars=10 μm.

Figure S9. Immunofluorescence of unmodified BSA in fixed BV-2 cells.

Conditions were identical to those in **Fig. 2b**, except that BV-2 cells were fixed, and stained with the F-actin dye Phalloidin, and the nuclear stain propidium iodide (PI) 24 h after incubation with unmodified BSA. Fluorescence signals for F-actin (green) and nuclei (red) were detected using a confocal microscopy. Bars=10 μm.

Supplementary Data 2

Materials and methods

1. MDA preparation and BSA modification by malondialdehyde - Bovine serum albumin (BSA) was purchased from Amresco (USA). MDA was prepared as described earlier (Li et al., 2010). In brief, fresh MDA was made by hydrolyzing 1,1,3,3-tetramethoxypropane (TMP) (purity ≥ 98%, Fluka Chemie AG, Buchs, Switzerland) and dissolved in phosphate buffer (PBS, pH 7.4) to make a stock solution of 200 mM (4.05% w/w) (Xu et al., 2012). BSA was dissolved in 10 mM PBS buffer (pH 7.4) (final concentration 45 μM) and incubated with malondialdehyde (final concentration 12 mM) at 37°C for up to 168 h. The MDA-modified BSA (mBSA) was washed four times by ultra-filtration to remove free MDA. MDA-modified BSA was retained by size exclusion centrifugation using Amicon Ultra-4 tubes (Millipore, USA).

2. Transmission electron microscopy (TEM) - BSA (1.5 μM) conjugated to MDA at different length of time was dripped onto formvar-coated EM grids (5 μl), and mBSA solution was removed with filter paper after 1 min. Samples were washed twice with ddH_2O and soaked in 3% uranyl acetate for 30 sec. Protein particles of BSA alone (control) and mBSA were visualized using a Philip Tecnai 20 Electron Microscope (FEI Co., Eindhoven, Netherlands) equipped with a digital camera.

3. Fluorescence analysis - Fluorescence measurements were performed using a

Hitachi F-4500 fluorescence spectrophotometer (Japan). Protein (0.25 μM) in 10 mM PBS buffer (pH 7.4) was used for all measurements unless stated otherwise. Fluorescence measurements were repeated at least three times for each sample. The fluorescence of MDA-modified BSA (λ_{ex}=400 nm, λ_{em}=465 nm) was measured at 37°C. MDA-modified BSA (0.5 μM) was used to measure the (OD_{280nm} < 0.01) fluorescence quantum yields on a Fluorolog-3 spectrofluorometer (HORIBA Jobin Yvon, Japan). Subsequently, BSA incubated with or without MDA for 48 h was used for cell experiments.

4. Cell culture - The immortalized microglial cell line (BV-2) was purchased from the National Platform of Experimental Cell Resources for Sci-Tech (China). Cells were grown in Dulbecco's Modified Eagle's Medium (DMEM, Life Technologies, USA) supplemented with 10% fetal bovine serum (FBS, PAA, Austria) and 1% penicillin/streptomycin in a humidified atmosphere of 5% CO_2 at 37°C.

5. Cell viability assay - Cell viability was determined using a 3-[4,5-dimethylthiazol-2-yl]-2,5-diphenyltetrazolium bromide (MTT) reduction assay (Sigma, USA). Cells (1×10^4 cells/ml) were seeded into poly-l-lysine-coated 96-well plates (0.1 ml/well), and incubated for 24 h in mBSA of different concentrations. Control cells were incubated with the corresponding concentrations of unmodified BSA. Subsequently, cell viability was analyzed using the MTT reduction assay (Liu et al., 2011). Cells were treated with 0.5 mg/ml MTT for 4 h prior to exposure to 100% dimethylsulfoxide for 10 min. The optical density (OD) of the dissolved formazan grains within the cells was measured spectrophotometrically at 490 nm. The viability of control cells was set to 1, and the percentage of viable cells collected from each treatment was calculated relative to the control group.

6. MDA-treatments and immunocytochemistry - Cells were seeded onto poly-l-lysine-coated coverslips positioned inside wells of 24-well plates at a concentration of 1×10^5 cells/ml (1 ml). After 12 h of culturing, medium was replaced

with fresh DMEM containing 7.5 μM MDA-modified BSA or 7.5 μM unmodified BSA, and cells incubated for 24 h. Subsequently, cells were fixed in 4% paraformaldehyde and permeabilized in 0.1% Triton X-100. Cells were incubated with F-actin dye Phalloidin (Life Technology, USA) at room temperature for 20 min. After three 5min washes in PBS, nuclei were stained with propidium iodide (PI, 1:1000 dilution, Beyotime, China). Finally, coverslips were mounted onto slides in GVA mounting solution (Zhongshan Goldbridge Biotechnology, China). Immunocytochemistry imaging was performed using a laser scanning confocal microscopy (LSCMFV500, Olympus, Japan) with a 60×/NA 1.4 objective. Images were taken using a 2048 × 2048 pixel EM-CCD camera mounted to the LSCM. Data acquisition and analysis were performed with the FV10-ASW software.

7. Analysis of live cells - 1.5×10^3 BV-2 cells were seeded into a confocal dish (cover-glass bottom dish, Corning, USA). After culturing for 12 h, cells were treated with 7.5 μM MDA-modified BSA. BV-2 cells treated for different periods of time were stained with LysoTracker, a lysosomal dye for live cells with an emission maximum at 590 nm (Life Technologies, USA). After 2 h, the staining medium was replaced by fresh DMEM, and cells were immediately analyzed by LSCM (LSCMFV500, Olympus, Japan). The imaging conditions used were identical to those used for immunocytochemistry imaging. Untreated BV-2 cells and cells treated with α-synuclein were used as controls.

8. Data analysis - All values are shown as means ± standard errors (SE) unless otherwise indicated. Data analysis was performed by one way analysis of variance (ANOVA) using Origin 8.0 statistical software. Differences were considered to be statistically significant at 5% ($p<0.05$).

Supplementary Data 3

The advantages for MDA as a fluorescent probe *in vivo* and *in vitro*.

In this study, we found that MDA modification not only induces BSA polymerization, but also endows BSA with a new fluorophore that can emit fluorescent light at an emission peak of around 465 nm. Because its maximum emission wavelength is located in the blue section of visible light, it does not overlap with those of other frequently used bio-molecules with fluorescence in the red, yellow and green color regions of the light spectrum. Using mBSA as a fluorescent probe, we demonstrated the translocation of mBSA into live microglia cell line BV-2 cells. Furthermore, we observed that malondialdehyde also reacts with other proteins, as tested with alpha-synuclein. Thus, the fluorescent protein adducts have the potential to be used as probes *in vivo* and *in vitro*.

MDA adducts as fluorescent probes have the following properties: (1) The fluorescence intensity is very strong, with a quantum yield of 0.163. (2) MDA itself exhibits a very low fluorescence background, but the emission becomes much stronger when the compound reacts with a protein, as observed for both BSA and AS. This is very convenient, as the signal of MDA-modified proteins is clearly distinguishable from any background signal originating from MDA itself. (3) Because of the strong emission, MDA modified proteins can be visualized conveniently on a SDS-PAGE gel using UV-light, without the need for Coomassie brilliant blue staining, resembling EB staining of DNA. (4) MDA derivatives can be used in live cell staining, as shown in the present study, thanks to low levels of cytotoxicity at concentrations required for appropriate fluorescent signal. (5) It also has a strong and distinguishable signal when co-stained with F-actin dye Phalloidin (green) in cell, as well as the nucleic probe PI (red).

Increased oxidative stress and formaldehyde stress play significant role in the etiology of cardiovascular disease, diabetes and neurodegeneration (He et al., 2010). Lipid peroxidation, which is initiated in the presence of hydroxy radicals and results in the production of malondialdehyde, directly produces oxidative stress. Thus,

tracing MDA-modified proteins by detecting their fluorescence is of great potential as a convenient method to investigate oxidative stress, in particular during lipid peroxidation observed in related diseases.

References

He, R., Lu, J., and Miao, J. (2010). Formaldehyde stress. Sci China Life Sci 53, 1399-1404.

Li, F., Yang, Z., Lu, Y., Wei, Y., Wang, J., Yin, D., and He, R. (2010). Malondialdehyde suppresses cerebral function by breaking homeostasis between excitation and inhibition in turtle Trachemys scripta. PLoS One 5, e15325.

Liu, Y., Qiang, M., Wei, Y., and He, R. (2011). A novel molecular mechanism for nitrated {alpha}-synuclein-induced cell death. J Mol Cell Biol 3, 239-249.

Xu, Y.J., Qiang, M., Zhang, J.L., Liu, Y., and He, R.Q. (2012). Reactive carbonyl compounds (RCCs) cause aggregation and dysfunction of fibrinogen. Protein Cell 3, 627-640.
