# Supplementary material for: Autofluorescence of MDA-modified proteins as an in vitro and in vivo probe in oxidative stress analysis
Source: Protein Cell. 2014 Apr 3;5(6):484–7. doi: 10.1007/s13238-014-0052-1 (PMC4026423; doi:10.1007/s13238-014-0052-1)
Supplement: Supplementary file 2 — Supplementary material 2 (PDF 983 kb) [file 13238_2014_52_MOESM2_ESM.pdf]

Fig. S1 Qiang et. al

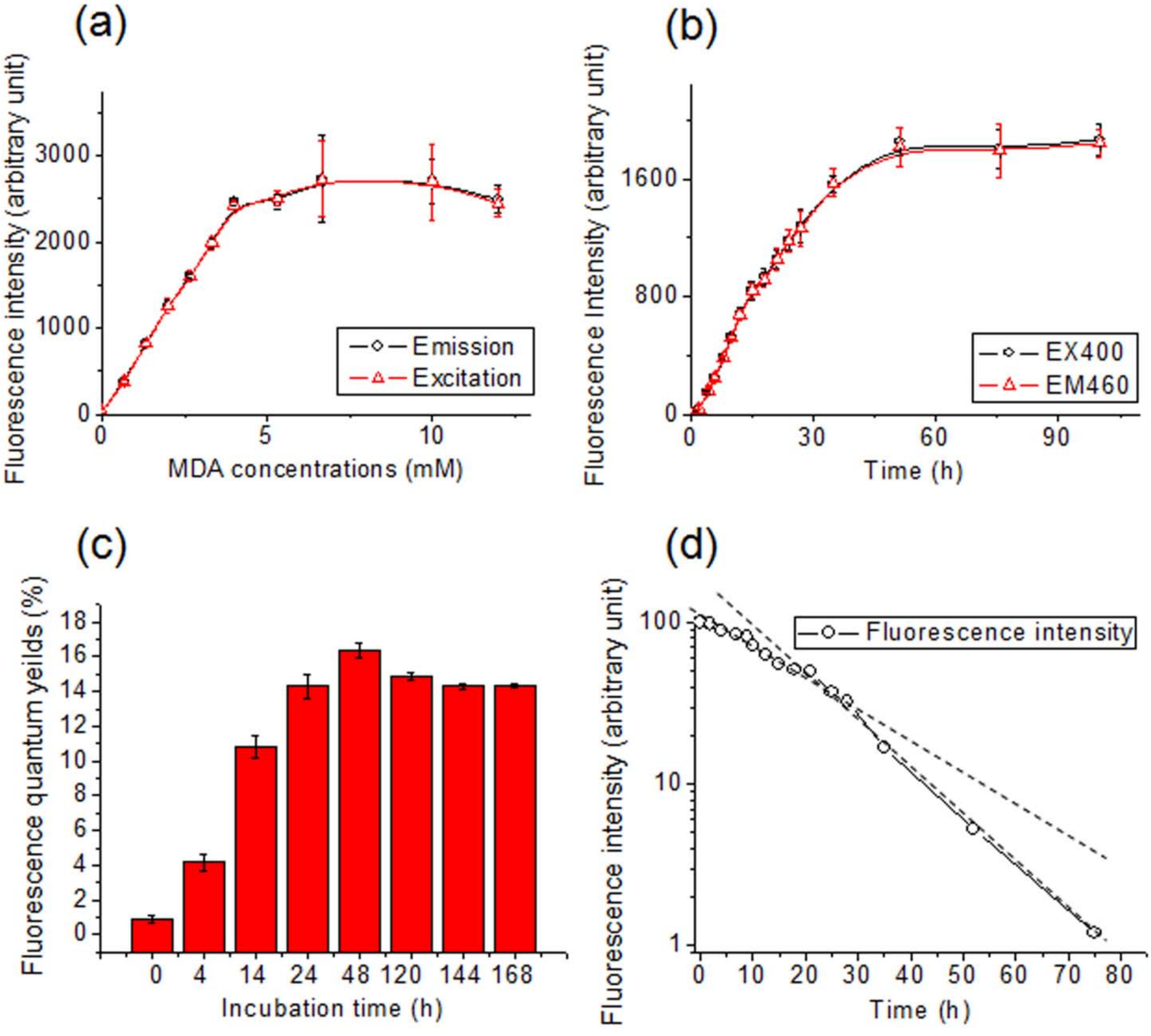

Fig. S2. Qiang et al.

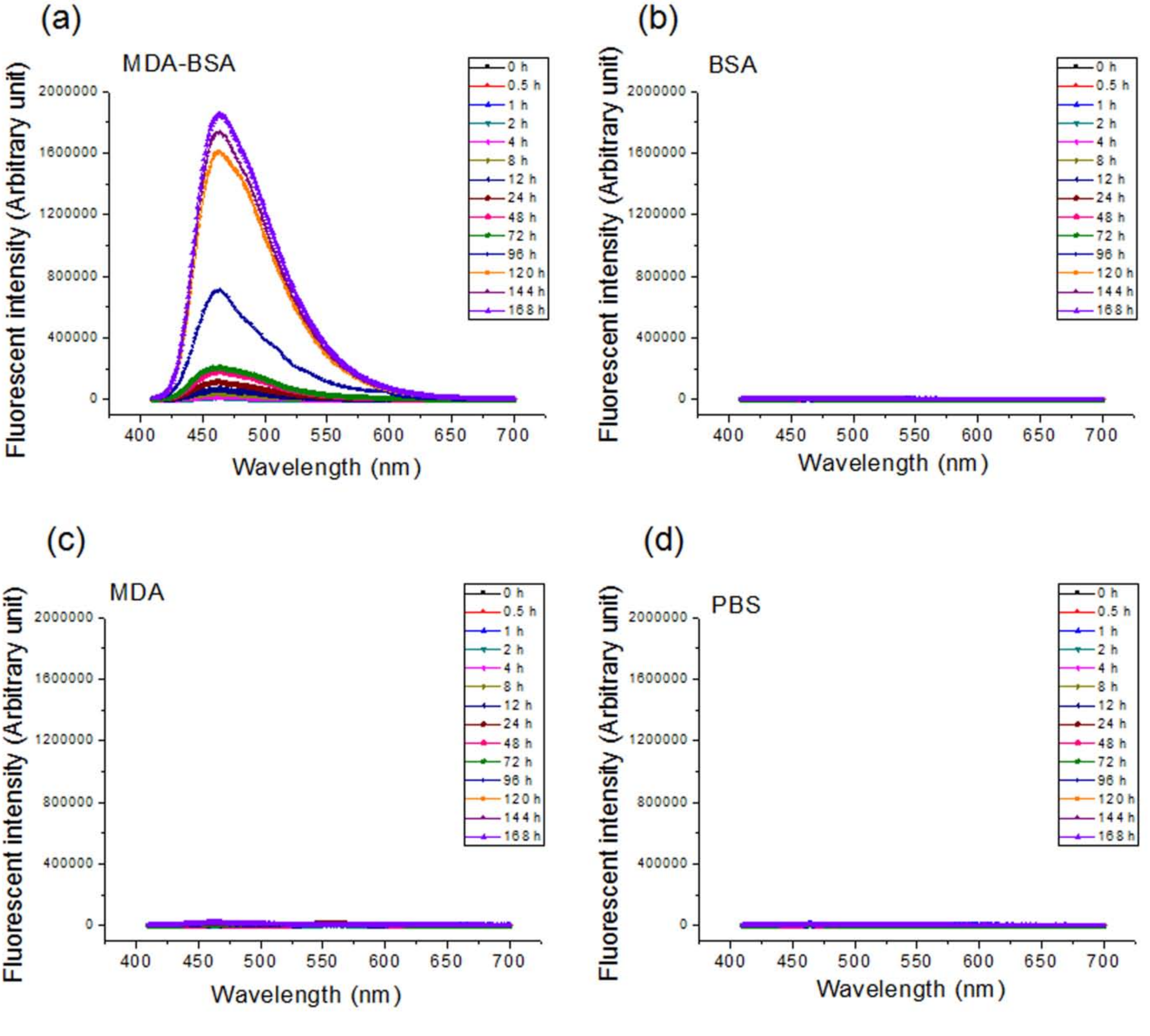

Fig. S3 Qiang et. al

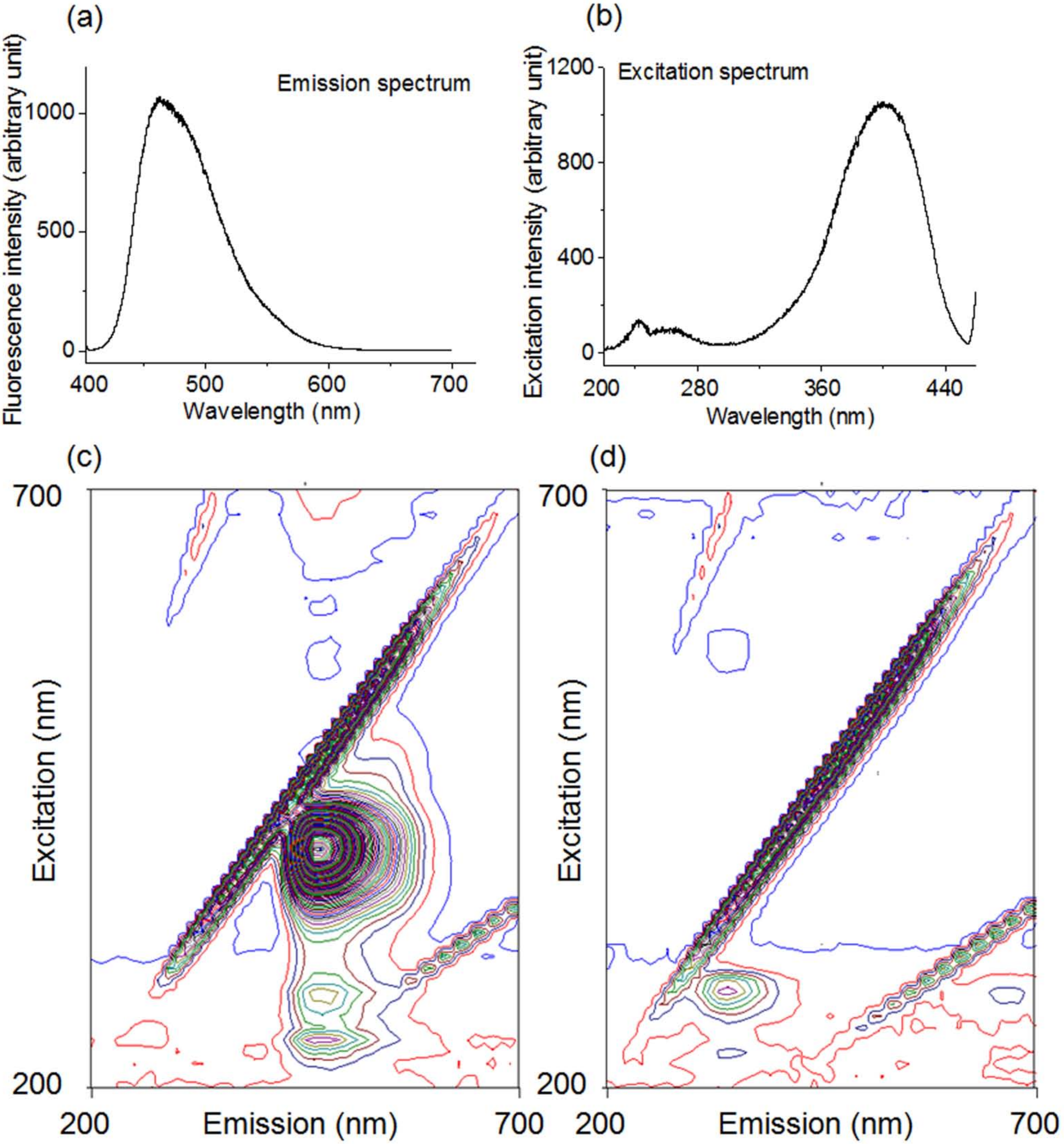

Fig. S4 Qiang et. al

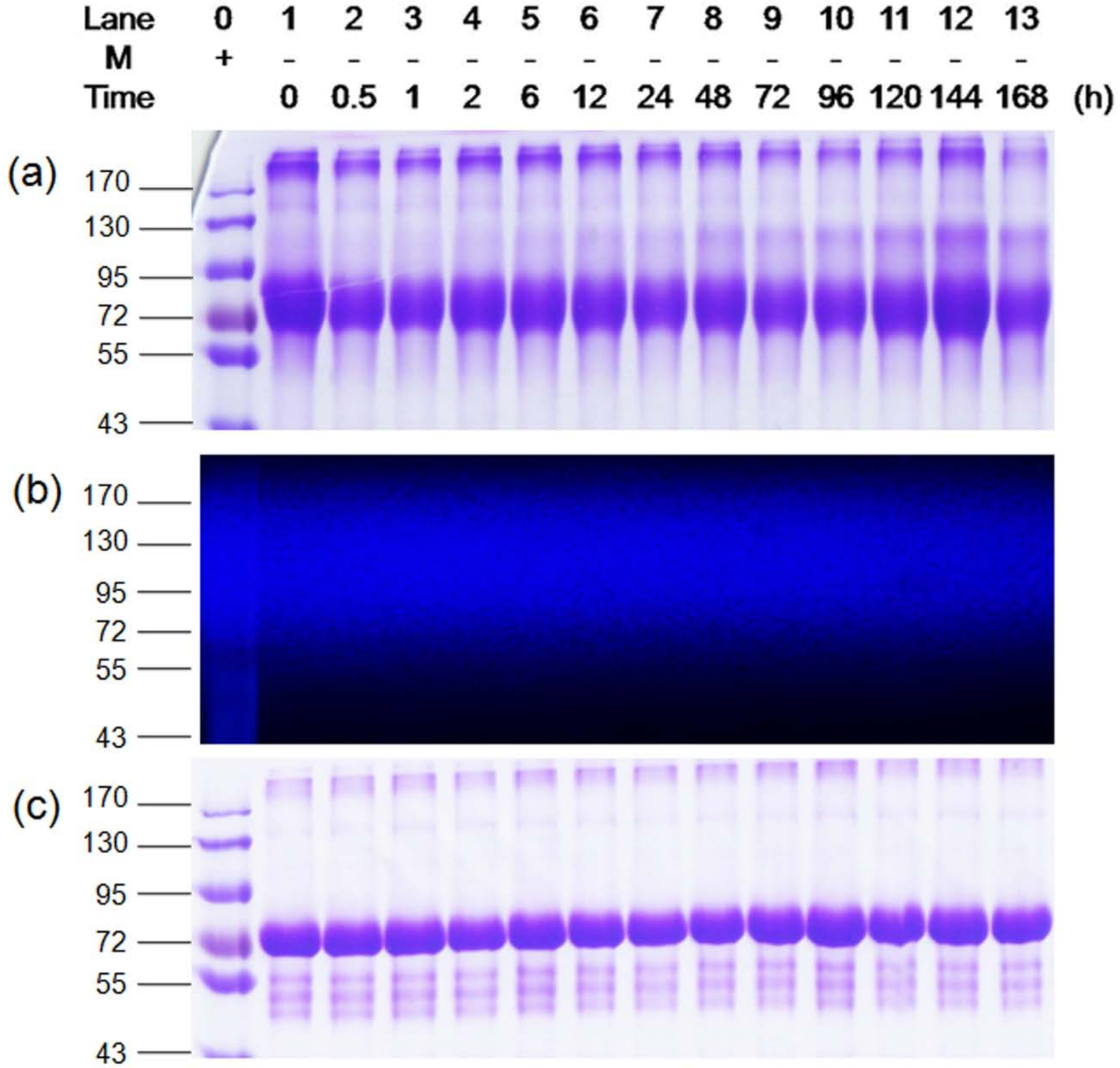

Fig. S5. Qiang et al.

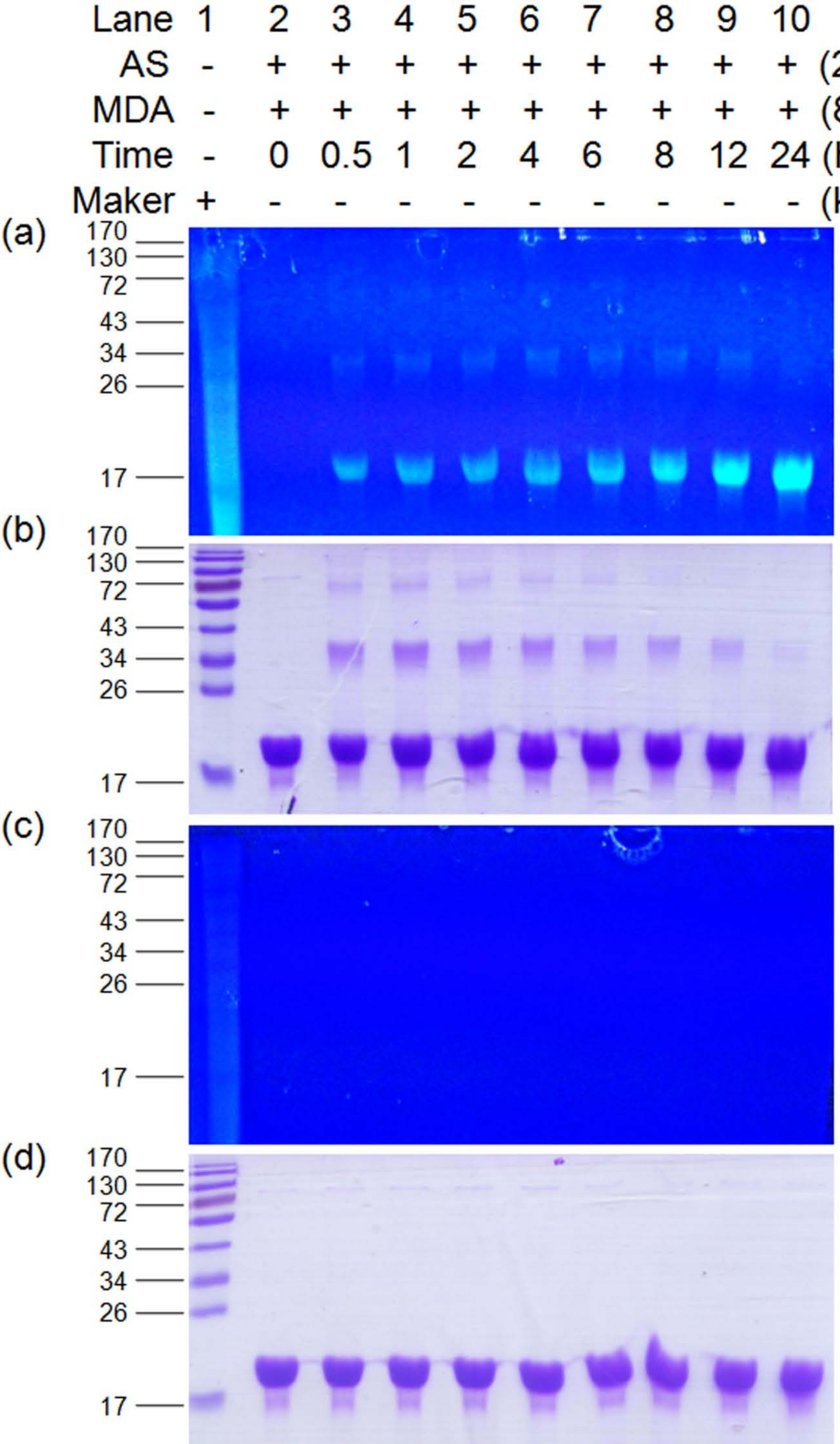

Fig. S6 Qiang et. al

(a)

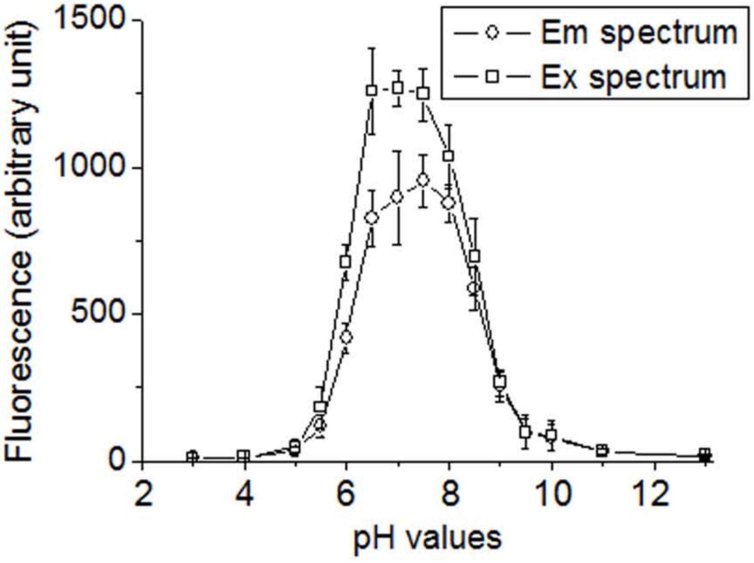

(b)

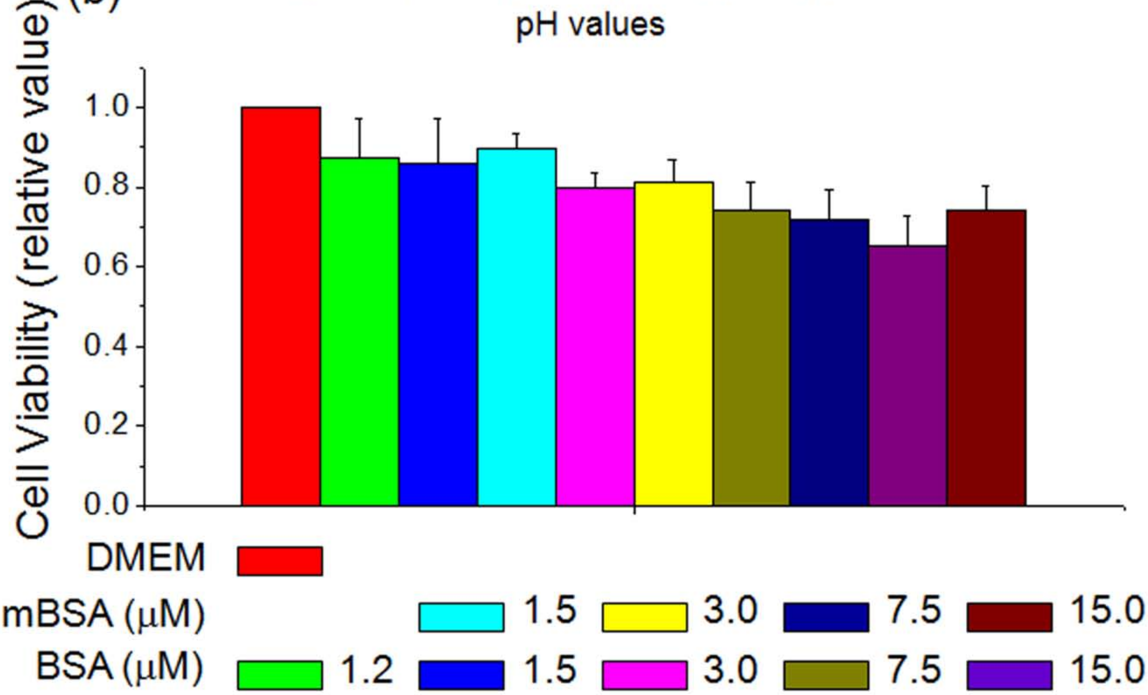

Fig. S7 Qiang et. al

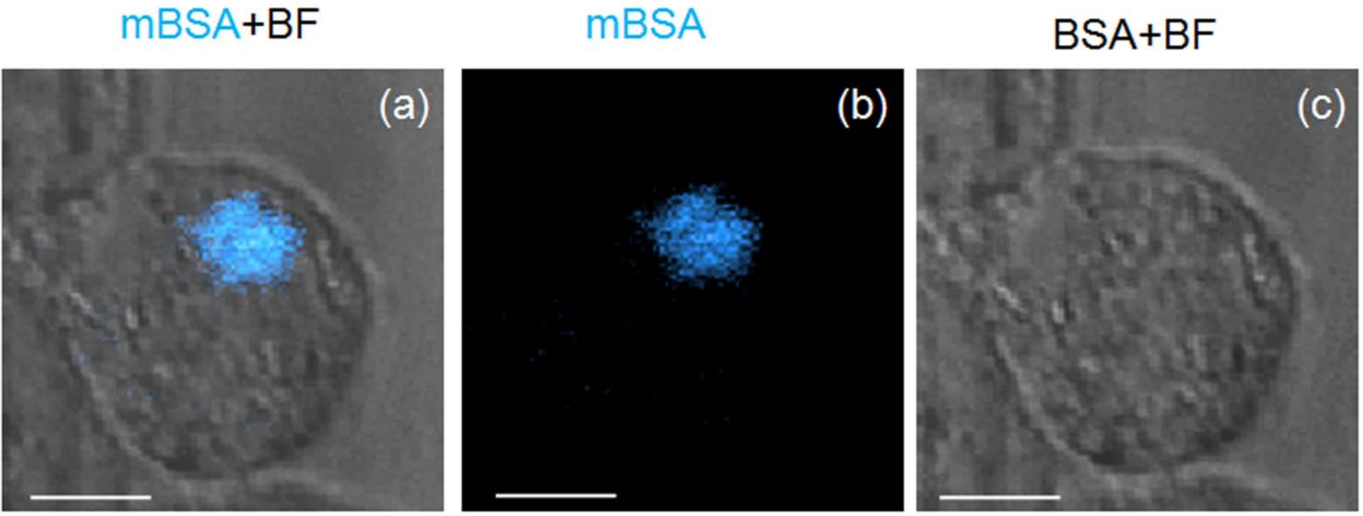

Bars = 10 μm

Fig. S8. Qiang et al.

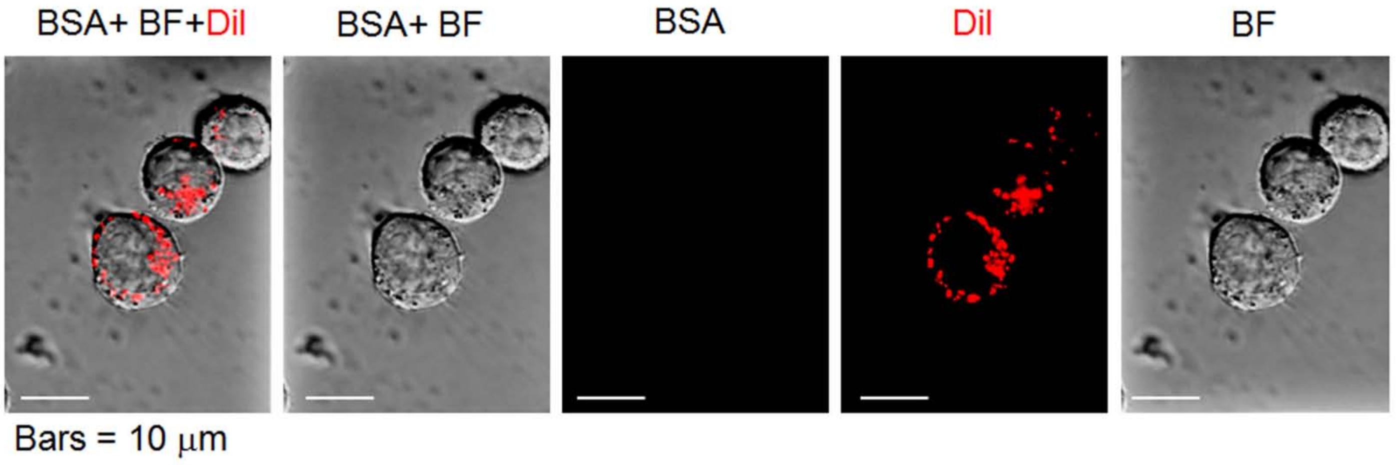

Fig. S9 Qiang et. al

BSA+PI+F-actin　　BSA+PI+F-actin　　BSA+F-actin　　BSA

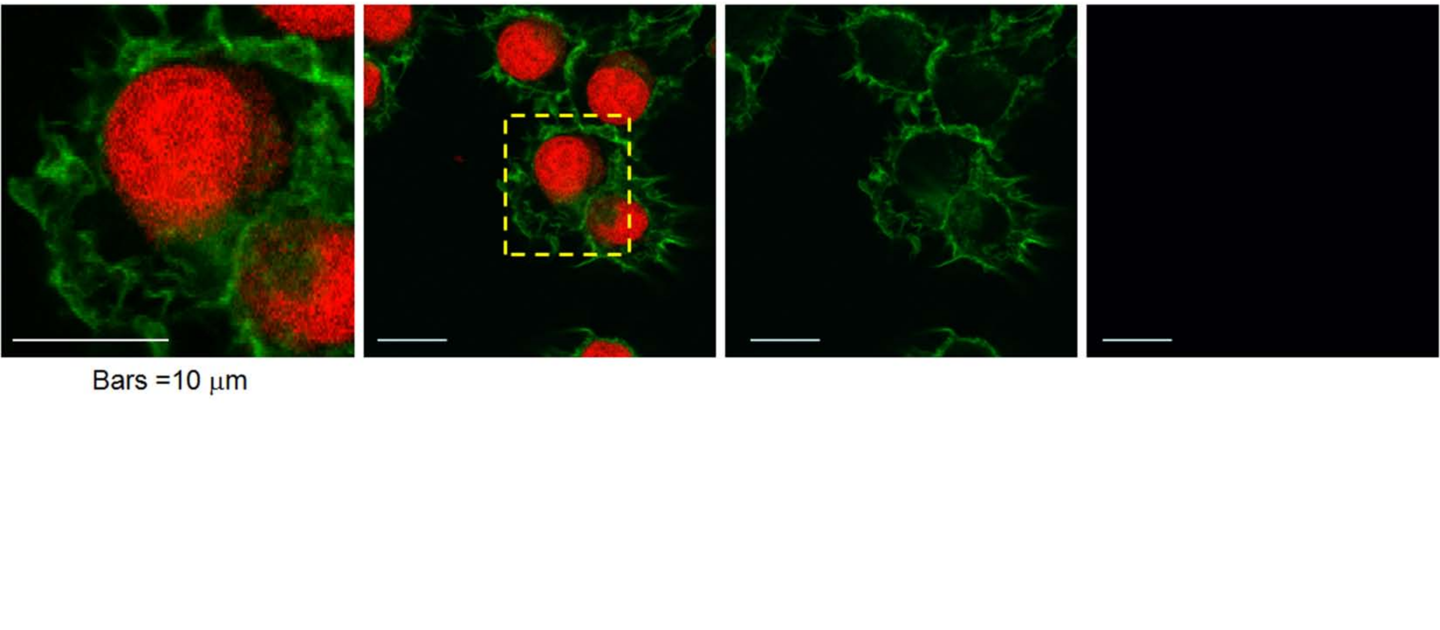

Bars =10 μm
